# Collaboration networks of the implementation science centers for cancer control: a social network analysis

**DOI:** 10.1186/s43058-022-00290-6

**Published:** 2022-04-13

**Authors:** Rebekah R. Jacob, Ariella R. Korn, Grace C. Huang, Douglas Easterling, Daniel A. Gundersen, Shoba Ramanadhan, Thuy Vu, Heather Angier, Ross C. Brownson, Debra Haire-Joshu, April Y. Oh, Robert Schnoll

**Affiliations:** 1 Prevention Research Center in St. Louis, Brown School, Washington University in St. Louis, One Brookings Drive, Campus Box 1196, St. Louis, MO 63130 USA; 2 Cancer Prevention Fellowship Program, Implementation Science, Office of the Director, Division of Cancer Control and Population Sciences, National Cancer Institute, 9609 Medical Center Drive, Rockville, MD 20850 USA; 3 Westat, 1600 Research Blvd., Rockville, MD 20850 USA; 4 Department of Social Sciences and Health Policy, Wake Forest School of Medicine, Winston-Salem, NC 27157 USA; 5 Dana-Farber Cancer Institute, Division of Population Sciences, 450 Brookline Avenue, Boston, MA 02215 USA; 6 Department of Social and Behavioral Sciences, Harvard T.H. Chan School of Public Health, Boston, MA 02115 USA; 7 Health Promotion Research Center, Department of Health Systems and Population Health, University of Washington School of Public Health, Seattle, WA 98195 USA; 8 Department of Family Medicine, Oregon Health & Science University, 3181 SW Sam Jackson Park Road, Portland, OR 97239 USA; 9 Department of Surgery (Division of Public Health Sciences) and Alvin J. Siteman Cancer Center, Washington University School of Medicine, Washington University in St. Louis, St. Louis, Missouri 63130 USA; 10 Center for Diabetes Translation Research, Washington University in St. Louis, 1 Brookings Drive, Campus Box 1196, St. Louis, MO 63117 USA; 11 Department of Medicine, Washington University School of Medicine, Washington University in St. Louis, St. Louis, MO 63130 USA; 12 Division of Cancer Control and Population Sciences, Implementation Science Team, National Cancer Institute, National Institutes of Health, 9609 Medical Center Drive, Rockville, MD 20850 USA; 13 Department of Psychiatry and Abramson Cancer Center, University of Pennsylvania, 3535 Market Street, 4th Floor, Philadelphia, PA 19104 USA

**Keywords:** Dissemination and implementation, Scientific collaboration, Evaluation, Cancer control, Social network analysis

## Abstract

**Background:**

Multi-center research initiatives offer opportunities to develop and strengthen connections among researchers. These initiatives often have goals of increased scientific collaboration which can be examined using social network analysis.

**Methods:**

The National Cancer Institute (NCI)-funded Implementation Science Centers in Cancer Control (ISC^3^) initiative conducted an online social network survey in its first year of funding (2020) to (1) establish baseline network measures including the extent of cross-center collaboration and (2) assess factors associated with a network member’s access to the network such as one’s implementation science (IS) expertise. Members of the seven funded centers and NCI program staff identified collaborations in *planning/conducting research*, *capacity building*, *product development*, *scientific dissemination*, *and practice/policy dissemination*.

**Results:**

Of the 192 invitees, 182 network members completed the survey (95%). The most prevalent roles were faculty (60%) and research staff (24%). Almost one-quarter (23%) of members reported advanced expertise in IS, 42% intermediate, and 35% beginner. Most members were female (69%) and white (79%). One-third (33%) of collaboration ties were among members from different centers. Across all collaboration activities, the network had a density of 14%, suggesting moderate cohesion. Degree centralization (0.33) and betweenness centralization (0.07) measures suggest a fairly dispersed network (no single or few central member(s) holding all connections). The most prevalent and densely connected collaboration was in *planning/conducting research* (1470 ties; 8% density). *Practice/policy dissemination* had the fewest collaboration, lowest density (284 ties’ 3% density), and the largest number of non-connected members (*n*=43). Access to the ISC^3^ network varied significantly depending on members’ level of IS expertise, role within the network, and racial/ethnic background. Across all collaboration activities, most connected members included those with advanced IS expertise, faculty and NCI staff, and Hispanic or Latino and white members.

**Conclusions:**

Results establish a baseline for assessing the growth of cross-center collaborations, highlighting specific areas in need of particular growth in network collaborations such as increasing engagement of racial and ethnic minorities and trainees or those with less expertise in IS.

**Supplementary Information:**

The online version contains supplementary material available at 10.1186/s43058-022-00290-6.

Contributions to the literature We conducted a social network analysis of the NCI-funded Implementation Science Centers in Cancer Control initiative to establish baseline network measures to track network growth and identify target areas for network interventions. The resulting snapshot is important to the implementation science field because of the following:

- Increasing network cohesion affects how we “do business” as researchers, cross-pollinating, and likely speeding the production, dissemination, and adoption of scientific findings.
- Determining network actions to better engage disconnected and under-represented members can guide other initiatives.
- Using network data as an evaluation tool can be an effective way to understand the processes involved in enhancing scientific collaboration.

## Background

In 2018, the National Cancer Institute (NCI) issued requests for funding to support Implementation Science Centers for Cancer Control (ISC^3^) (2019-2024) [1–3]. ISC^3^ aims to build scientific capacity in the field with targeted approaches for developing and testing innovative methods and measures for dissemination and implementation research while engaging scholars in a rich network of investigators [3]. Collaboration among ISC^3^ investigators and staff within and across centers is critical and can lead to greater productivity and impact, diverse thinking, and increased opportunities for capacity building in the field [4–6]. Priming the network to develop additional scientific linkages between researchers is a key focus of the ISC^3^ and, therefore, understanding the extent of these connections is an important evaluation priority.

Social network analysis is a useful methodology for evaluating multi-center initiatives such as ISC^3^ that aim to build and expand networks and enhance network cohesion [7–13]. Understanding linkages within networks informs areas where growth or additional types of collaboration are desired, which in turn can inform purposefully designed network interventions [14, 15]. This is especially important at the beginning of an initiative given the time to implement and measure outcomes resulting from the creation of new collaborations or other changes to linkages [15].

The purpose of this report is to describe the scientific collaborations and early linkages within the ISC^3^ network during the initiative’s first year, specifically in *planning or conducting research*, *capacity building*, *product development*, *scientific dissemination*, and *practice/policy dissemination* and to assess which factors are associated with those linkages. These data serve to aid in two evaluation aims (1) to characterize the network at baseline, especially with regard to cross-center collaboration and (2) to examine how network membership and connectedness vary by one’s role in the initiative, level of implementation science (IS) experience, and race/ethnicity. Assessing the network at baseline is essential to evaluating change over time while identifying variation in network membership informs ISC3’s agenda for increasing equity.

## Methods

ISC^3^ is made up of seven centers: Harvard T.H. Chan School of Public Health, Oregon Health & Science University, University of Colorado School of Medicine, University of Washington, Wake Forest School of Medicine and University of Massachusetts Medical School, Washington University in St. Louis, and the University of Pennsylvania. Funding started in October 2019 except for the University of Pennsylvania ISC^3^, which entered as a new center in October 2020. Additional information on each center can be found at https://cancercontrol.cancer.gov/is/initiatives/isc3.

In Fall 2019, the ISC^3^ Cross-Center Evaluation Workgroup developed a survey to assess intra- and inter-center research collaborations. Each center’s leadership team provided a list of faculty, staff, trainees, and others who were critical to their scope of work. Across the seven ISC^3^ centers and NCI’s program staff and leadership for the initiative, a total of 192 individuals (range 11–51/center) were invited to participate in the 10–25-min web-based survey in September 2020. The survey remained open for 6 weeks. This study met exemption status as it did not meet the criteria needed for human subjects research by Westat Institutional Review Board (No. 00005551).

### Measures

The full survey is provided in Additional file 1. Participants identified their direct contacts (i.e., via meetings, phone, and email) within the past 12 months across the roster of all 192 invited individuals. For each direct contact, participants selected collaborations in (1) *planning/conducting research* (e.g., grant writing, study design or execution), (2) *capacity building* (e.g., trainings, learning communities, mentoring), (3) *product development* (e.g., measures database, survey instrument, and other products from workgroups), (4) *scientific dissemination* (e.g., scholarly publication, conference presentation to scientific audience), and (5) *practice/policy dissemination* (e.g., evaluation report, policy brief to non-science audience). Participants also identified their scientific discipline, length of years working in their field, role within the ISC^3^ initiative, level of IS expertise, gender identity, and racial and ethnic background.

### Analysis

Network data were cleaned and analyzed using R with the *igraph* package [16]. Survey non-respondents who were nominated as collaborators by responders were included in the analyses. Ties were symmetrized for undirected analysis, a common approach for networks where the relational direction is not a major focus and collaboration is assumed from either direction [10, 17].

For aim 1, we explored the network structure visually (graphs) and descriptively across the five separate collaborative areas individually and then combined. We calculated density, degree centralization, betweenness centralization, transitivity, number of isolates, and proportion of collaborations within and across centers.

For aim 2, we assessed members’ connectedness (or access) to the network based on participant characteristics. We calculated degree centrality or the number of connections for each member by collaboration type. We examined median degree because tie data were skewed and extreme cases influenced the mean. We used Kruskal-Wallis chi-square tests to determine rank order differences in connectivity across categories of participant characteristics.

## Results

A total of 182 participants completed the survey (95% response rate; 91–100% across centers). ISC^3^ network member characteristics are reported in Table 1. Most participants reported their primary discipline as public health (54.4%) or medicine (24.2%). More than two thirds (69.8%) of members reported 10+ years of experience in their field. The most prevalent network roles were faculty (60.4%) and center staff (23.6%). The largest proportion of members reported intermediate (41.8%), followed by a beginner (35.2%) and advanced (23.1%) IS expertise. Most members identified as female (68.7%) and white (78.7%).

**Table 1:** Implementation Science Centers for Cancer Control (ISC^3^) Year 1 network participant characteristics (*n*=182)

Characteristic	Participant characteristics
	***n*** (%)
**Discipline**	
Public health	99 (54.4)
Medicine	44 (24.2)
Other^a^	39 (21.4)
**Experience in field**	
< 5 years	18 (9.9)
5–9 years	37 (20.3)
10–15 years	56 (30.8)
> 15 years	71 (39.0)
**Role**	
Trainee	12 (6.6)
Staff	43 (23.6)
Faculty	110 (60.4)
NCI staff	11 (6.0)
Other^b^	6 (3.3)
**Implementation science expertise level**	
Beginner	64 (35.2)
Intermediate	76 (41.8)
Advanced	42 (23.1)
**Gender identity**^c^	
Female	125 (68.7)
Male	56 (30.8)
**Racial/ethnic background**^d^	
White	140 (78.7)
Asian	18 (10.1)
Black or African American	11 (6.2)
Hispanic or Latino	5 (2.8)
Other	4 (2.2)

^a^Examples of other disciplines include psychology, social work, economics, health services research, and implementation science.

^b^Examples of other roles included consultants and advisors.

^c^*n*=181

^d^*n*=178

### Network characteristics

**Fig. 1:**
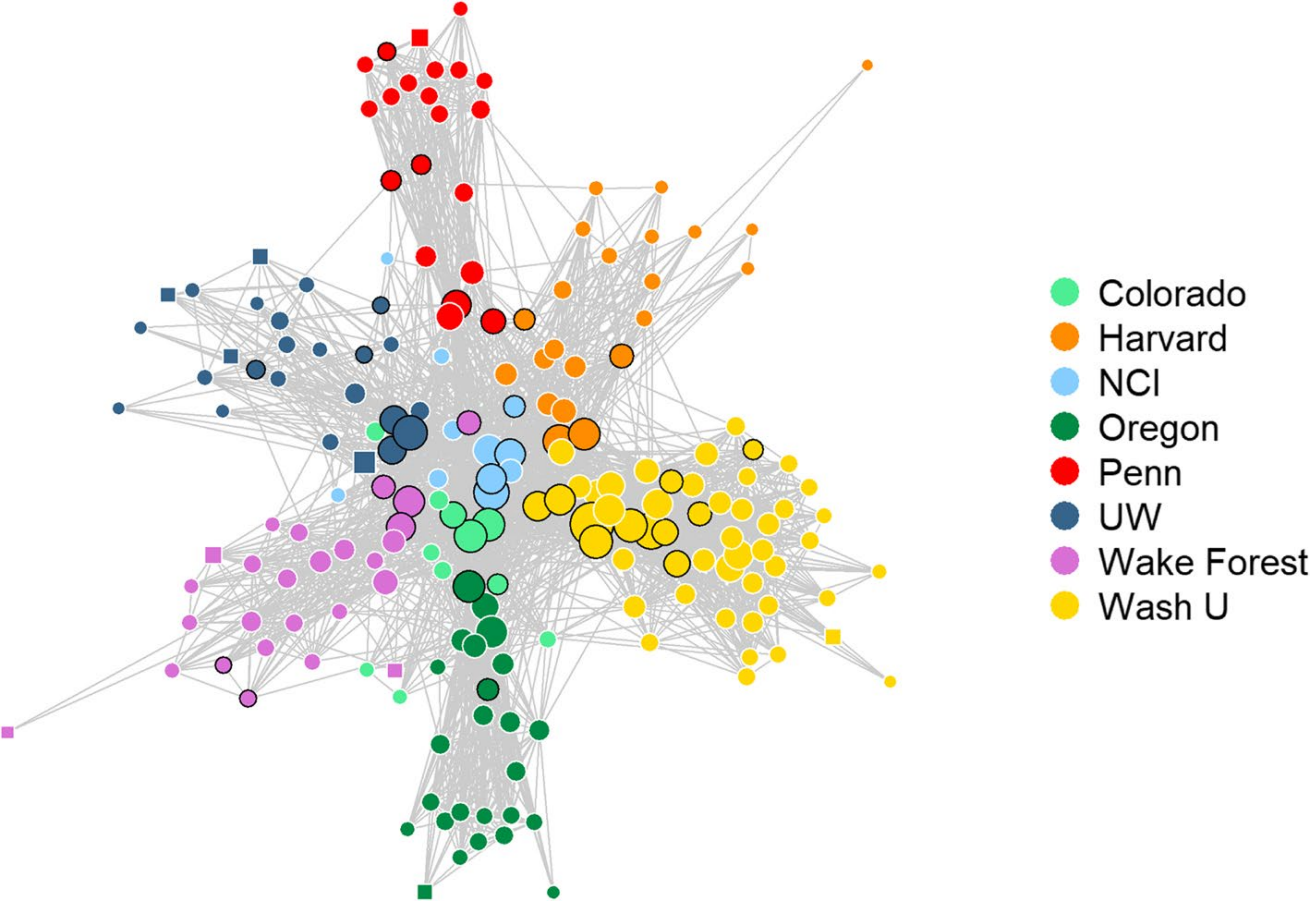
ISC^3^ network of all collaboration activities combined. Node color represents ISC^3^ center, node size represents degree centrality scores, and nodes with black borders indicate those reporting “advanced” expertise in implementation science. Square nodes represent those with missing information about IS expertise (*n*=10)

**Fig. 2:**
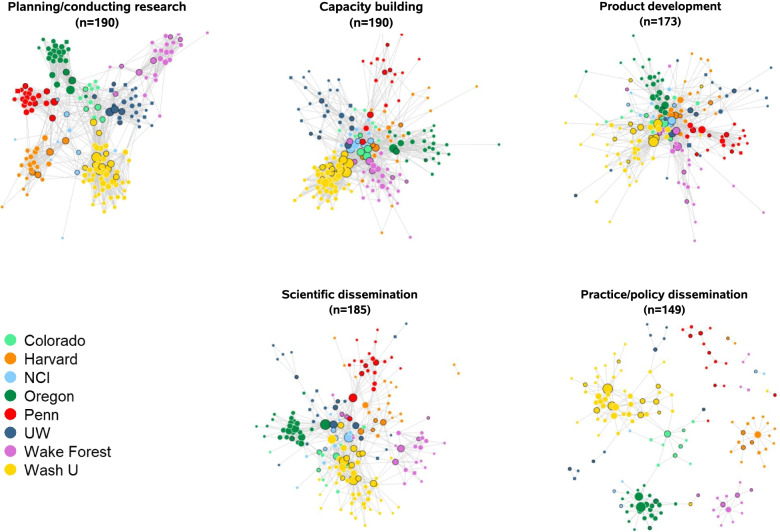
ISC^3^ collaborations in five network activities. Node color represents ISC^3^ center, node size represents degree centrality scores, and nodes with black borders indicate those reporting “advanced” expertise in implementation science. Square nodes represent those with missing information about IS expertise (*n*=10)

Ten survey non-respondents were nominated as collaborators by responders and included in the full network. With *all collaboration activities* combined, the ISC^3^ network included 192 members and 2480 collaboration ties, of which members had a median of 22 connections (Table 2). Figure 1 displays the network for *all collaboration activities* combined, and Fig. 2 displays the network for each collaboration type. The *practice/policy dissemination network* was the smallest network with 143 of the 192 ISC^3^ network members represented, whereas the other networks ranged from 173 to 190 members. The greatest number of ties were reported in *planning/conducting research* (1470 ties; median 15 ties/member), and the fewest ties were reported in *practice/policy dissemination* (284 ties; median 2 ties/member). For *all collaboration activities*, density, or the ratio of the number of ties to the total number of possible ties in the network, was 13.5%. Across the different collaboration types, the most and least densely connected networks were *planning/conducting research* (8.2%) and *practice/policy dissemination* (2.6%), respectively.

**Table 2:** Implementation Science Centers for Cancer Control (ISC^3^) Year 1 collaboration network descriptive characteristics

Network characteristic	All collaboration activities	Planning/conducting research	Capacity building	Product development	Scientific dissemination	Practice/policy dissemination
*N*	192	190	190	173	185	149
**Ties**	2480	1470	1336	825	654	284
% cross-center	33.0	11.7	31.0	48.1	23.5	6.0
**Median degree (range)**	22 (2, 89)	15 (1, 48)	10 (1, 58)	6 (1, 45)	5 (1, 30)	2 (1, 22)
Within-center	17 (2, 50)	13 (1, 44)	7 (1, 48)	4 (1, 25)	4 (1, 25)	2 (1, 21)
Cross-center	7 (1, 56)	3 (1, 17)	3 (1, 43)	5 (1, 40)	2 (1, 20)	1 (1, 4)
**Density (%)**^a^	13.5	8.2	7.4	5.5	3.8	2.6
**Betweenness centralization**^b^	0.07	0.12	0.13	0.11	0.23	0.20
**Degree centralization**^b^	0.33	0.17	0.23	0.21	0.12	0.12
**Transitivity**^c^	0.47	0.56	0.37	0.34	0.33	0.33
**Isolates**	0	2	2	19	7	43

*IS* implementation science, *NCI* = National Cancer Institute

^a^Density is the ratio of the number of ties to the total number of possible ties in the network; often used to measure the overall connectivity of a network or degree of cohesion among a network of collaborators [0, 1]

^b^Centralization is used to assess the extent of hierarchy in the network; extent that connections in the network are associated with a select few most central nodes in the network [0, 1]. Degree centralization is based on the number of connections (higher degree centralization=one or more nodes hold most of the connections), whereas betweenness centralization is used to measure the extent to which each network member represents a bridge or gatekeeper to others in the network (based on the number of connections or paths in the network an individual lies between, higher betweenness centralization=one or a few nodes responsible for holding the network together)

^c^Transitivity is a measure of clustering [0, 1] with higher transitivity suggests that new ties are more likely to form between nodes that share a common collaborator (e.g., referred by an existing collaborator)

The overall ISC^3^ network was fairly decentralized (degree centralization=0.33 and betweenness centralization=0.07; Table 2), consistent with Fig. 1’s basic linked local network shape (no strong central node or group of nodes). For separate activities, *capacity building* and *product development* had the highest degree of centralization (0.23 and 0.21, respectively) compared to other collaboration activities, which ranged from 0.12 to 0.17, suggesting influential positions for some members in these networks (“hub and spoke” network structure). *Scientific dissemination* and *practice/policy dissemination* networks had the highest betweenness centralization (0.23 and 0.20, respectively), suggesting that a smaller group of members keep the network connected in these activities.

Overall, the ISC^3^ network’s transitivity (0.47) suggests the heightened probability of triangles in the network, though variation exists across collaboration types. *Planning/conducting research* had the highest transitivity measure (0.56) compared to all other collaboration networks (transitivity range: 0.33 to 0.37), suggesting that two investigators that are collaborating with the same investigator are likely to also be collaborating with each other.

One-third of all collaboration ties (33.0%) occurred between members from different centers. We observed the largest proportion of cross-center collaboration in *product development* (48.1%), which includes involvement with cross-center workgroups. Collaboration on *practice/policy dissemination* and *planning/conducting research* mostly occurred within members’ respective centers (6.0% and 11.7% cross-center ties, respectively). Network members had a median of 17 connections within their center and 7 connections from other centers across all activities.

There were no isolates for the *all collaboration activities* network because our overall network was derived from having at least one collaboration activity reported. Notably, *practice/policy dissemination* and *product development* were the two activity networks with the largest number of isolates (*n*=43 and *n*=19, respectively). Half of the ISC^3^ trainees (*n*=6) were not connected to *product development*.

### Member connectedness by role, IS expertise, and racial/ethnic background

**Fig. 3:**
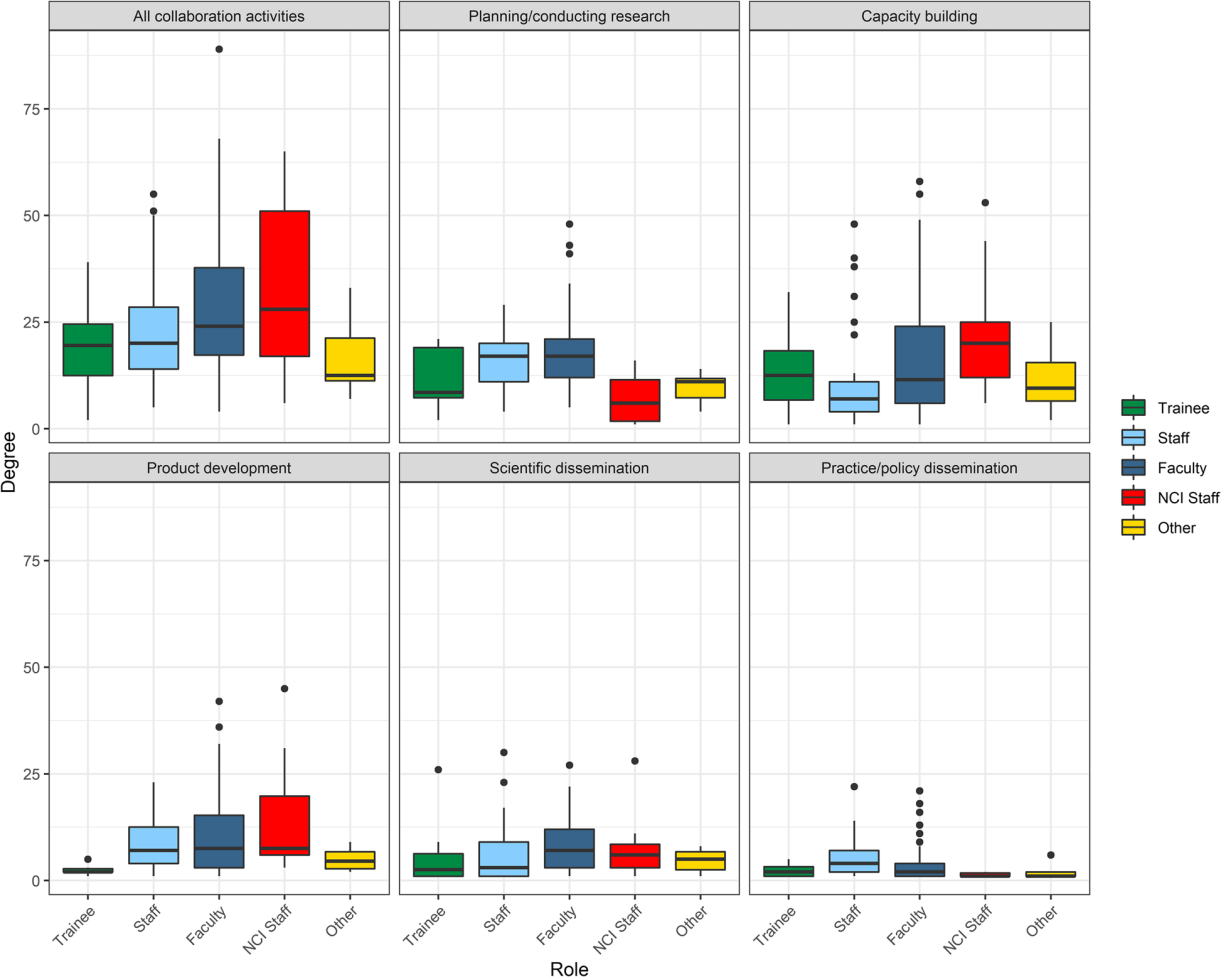
Degree distribution by member role. Each box represents the interquartile range with the median (black line) number of connections for each role group. Whiskers represent maximum and minimum connections without extreme outliers (separate black dots)

The number of connections (degree) varied significantly across ISC^3^ roles in *all collaboration activities* combined (*χ*^2^=10.59(4), *p*=0.032) with NCI staff having the highest median degree in all activities combined (28 (range: 6–65) ties), followed by faculty (24 (4–89) ties) (Fig. 3) (Additional file 2). Degree also varied significantly by role for *planning/conducting research* (*χ*^2^= 27.94(4), *p*=<0.001), *capacity building* (*χ*^2^=11.97(4), *p*=0.018), *product development* (*χ*^2^=10.06(4), *p*=0.039), *scientific dissemination* (*χ*^2^=11.31(4), *p*=0.023), and *practice/policy dissemination* (*χ*^2^=12.10(4), *p* = 0.017).

**Fig. 4:**
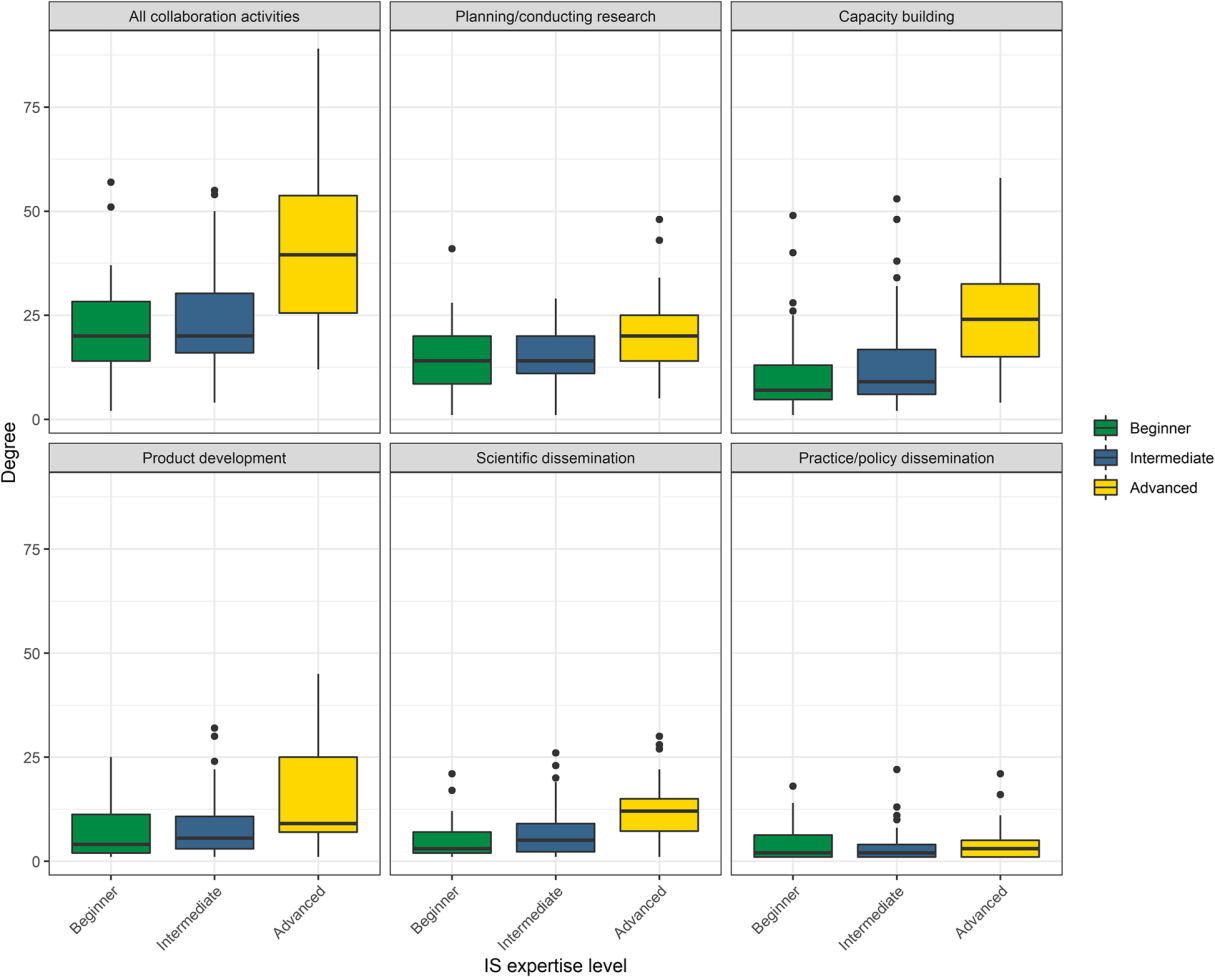
Degree distribution by member IS expertise. Each box represents the interquartile range with the median (black line) number of connections for each IS expertise group. Whiskers represent maximum and minimum connections without extreme outliers (separate black dots)

Members with advanced IS expertise were more connected in all networks (Additional file 2); these individuals are shown in Figs. 1 and 2 as nodes with a black border. Median degree varied significantly across IS expertise levels in *all collaboration activities* combined (*χ*^2^=34.42(2), *p*=<0.001) and in four of the five activity networks: *planning/conducting research* (*χ*^2^=15.74(2), *p*=<0.001), *capacity building* (*χ*^2^=34.17(2), *p*=<0.001), *product development* (*χ*^2^= 20.21(2), *p*=<0.001), and in *scientific dissemination* (*χ*^2^=40.80(2), *p*=<0.001) (Fig. 4).

**Fig. 5:**
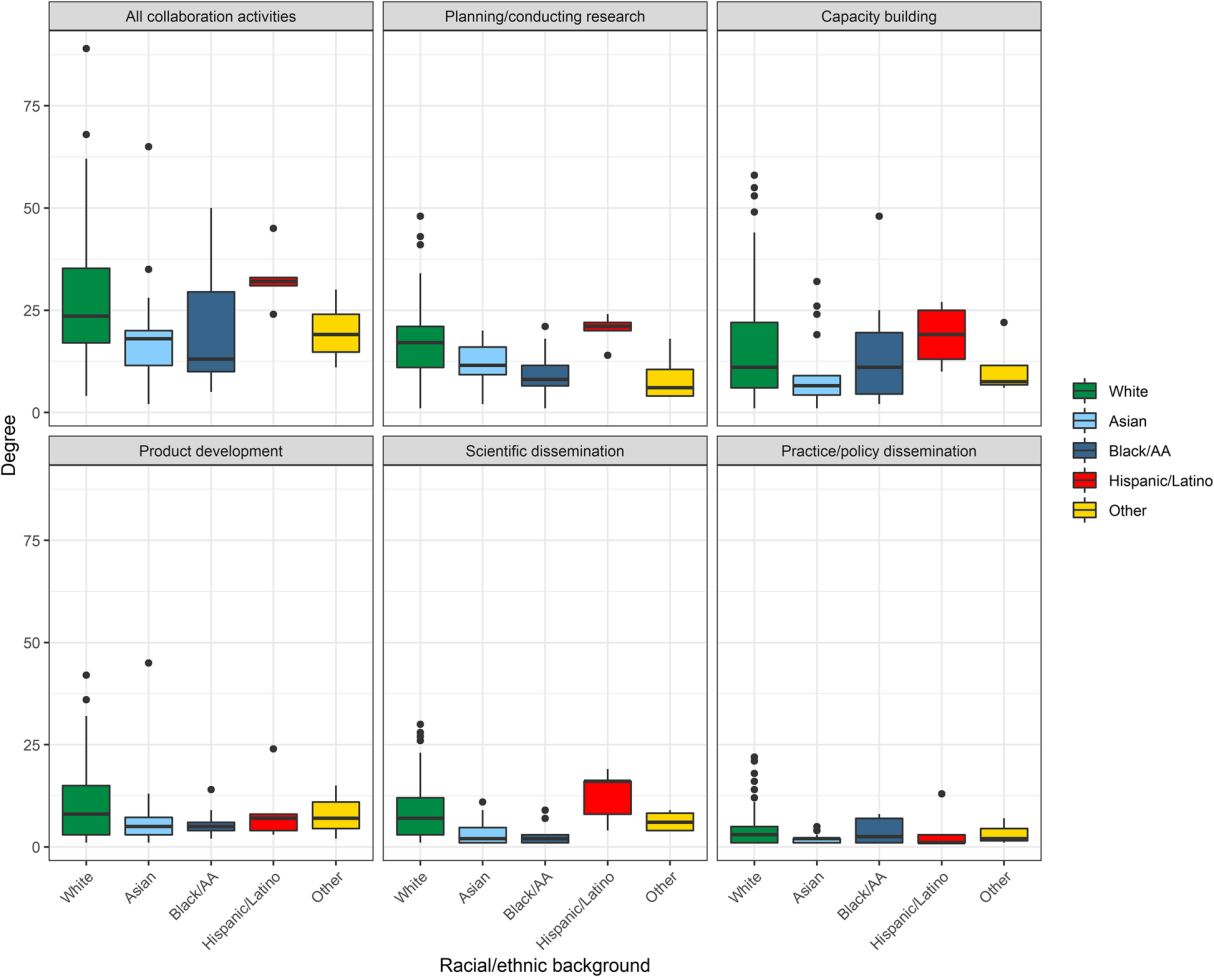
Degree distribution by member race/ethnicity. Each box represents the interquartile range with the median (black line) number of connections for each racial/ethnic group. Whiskers represent maximum and minimum connections without extreme outliers (separate black dots)

Degree varied significantly across racial/ethnic backgrounds in *all collaboration activities* combined (*χ*^2^=13.14(4), *p*=0.011), *planning/conducting research* (*χ*^2^=25.52(4), *p*=<0.001), *scientific dissemination* (*χ*^2^=22.50(4), *p*=<0.001) (Fig. 5)*.* Hispanic or Latino network members were most connected in all collaborations (32 (24–45) ties) followed by white members (23.5 (4–89) ties). Black or African-American members were least connected (13 (5-50) ties) (Additional file 2).

## Discussion

This report illustrates the range of insights offered by a network evaluation of a multi-center research initiative. The analysis highlights several opportunities to increase participation in cross-center and activity-specific networks. The early evaluation of network participation also provides centers with an opportunity to improve the identification, engagement, and retention of underrepresented groups, including racial minorities and trainees. A critical future evaluation direction is a comparison of network activity and growth as an outcome of the ISC^3^ initiative over the 5-year funding period.

In exploring the ISC^3^ network in its first year of funding, we established a baseline of inter/intra-center collaboration by which to gauge changes over time. The majority of collaborations were among members within the same center, though the level varied across centers. This likely reflects where several IS collaborations were already established prior to new funding. We expect that collaboration within centers will remain the same or increase over time. We also anticipate increases in cross-center collaboration given the robust participation in workgroups and other cross-center projects and activities. For example, dissemination and policy collaboration may increase as center-specific research or workgroup projects wind down and natural collaboration opportunities emerge such as disseminating findings via papers and policy recommendations, etc. Additionally, we expect the network to grow in size through funding of pilot studies, which introduce additional members with various expertise.

The results indicate areas of collaboration that may require specific support moving forward. Among network activities, *practice/policy dissemination* collaborations were sparse, with five-fold fewer ties than *planning/conducting research*. This could be due to investigators focusing on intra- and inter-network project planning in the first year of the new initiative that more generally precedes dissemination to non-science audiences. Even so, to enhance dissemination to audiences other than researchers, more attention is needed on designing for dissemination (in all stages of the initiative), or the “active process that helps to ensure that public health interventions, often evaluated by researchers, are developed in ways that match well with adopters’ needs, assets, and time frames [18, 19].” Several processes could collaboratively be developed and implemented across the ISC^3^ including participatory co-design, context and situation analysis, methods from marketing and business, communications and visual arts, and systems science [19].

Trainees and those with beginner IS expertise have lower access to collaboration in the ISC^3^ network. Mentoring the next generation of IS investigators is imperative to “grow the network younger” and to assure that early-career members have equal access to collaborative activities and can increase scientific production overall [20–23]. The ISC^3^ can increase access to the network by using post-doctoral funding streams to connect early investigators to the larger ISC^3^ network, providing a platform to connect with other peers in the IS field from several other universities. Currently, the ISC^3^ supports five workgroups that are specific places where purposeful engagement of trainees could connect them not only to network activities, but also to potential senior mentors outside of their respective centers. Many of the centers are also partnering junior investigators with more experienced investigators in the leadership of studies supported by the centers.

White network members had the highest representation in the network (79%) and were highly connected across network activities. While Hispanic or Latino network members were also highly connected, they made up <3% of the entire network. In general, Black, Hispanic or Latino, Native American, and other groups are under-represented in the ISC^3^ network. This points to the need for more efforts to assemble and engage a diverse set of network members. Additionally, offering specific opportunities, like research funding, has the potential to pair previously unconnected members with those who are more connected within the network [24]. The ISC^3^ developed a supplemental funding avenue to enhance IS health equity-focused research collaborations across the entire network. Such funding could be a promising mechanism to include less connected investigators/researchers and also promote cross-center collaborations with a focus on equity, thereby attracting a more diverse group of investigators. In our next wave of data collection (year 3 of the funding cycle), we will determine any changes in network representation by race/ethnicity.

While these findings inform strategies to enhance scientific linkages across the network, limitations should be noted. First, it is possible that not every network member is positioned or skilled to be involved with every activity that we identified and collected information on. Social network surveys are self-reported and can introduce some respondent bias, and symmetrizing ties has implications for both respondents who tend to over-report and those that under-report collaborations. It is also possible that we missed people in the network with our center-identified roster approach, though with guidance on inclusion criteria, we believe this was likely minimized.

## Conclusions

We presented baseline scientific linkages across a robust network of centers working in implementation science in cancer control. The centers are fairly cohesive and have considerable cross-center collaborations underway. Even so, this snapshot highlights parts of the network where linkages should grow for the ISC^3^ initiative to meet its objectives to increase the number of trainees, enhance practice and policy dissemination, and expand engagement among members from underrepresented minority groups. Targeted interventions within the network are the next steps with plans to use this study as a baseline to measure changes in the network over time.

## Supplementary Information

**Additional file 1.** The ISC^3^ Social Network Survey Tool.

**Additional file 2.** ISC^3^ Year 1 network collaborations.

## Data Availability

The datasets analyzed during the current study are available from the corresponding author upon reasonable request.

## Abbreviations

ISC^3^: Implementation Science Centers for Cancer Control
IS: Implementation Science
