# Supplementary material for: Collaboration networks of the implementation science centers for cancer control: a social network analysis
Source: Implement Sci Commun. 2022 Apr 13;3:41. doi: 10.1186/s43058-022-00290-6 (PMC9009020; doi:10.1186/s43058-022-00290-6)
Supplement: Supplementary file 1 — Additional file 1. The ISC3 Social Network Survey Tool. [file 43058_2022_290_MOESM1_ESM.docx]

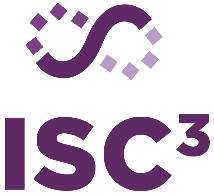


Scientific Collaboration Survey

### Introduction

The Implementation Science Centers in Cancer Control (ISC^3^) Evaluation Team invites you to help us describe the collaborations among the Implementation Science Centers in Cancer Control (ISC^3^) Program by completing this social network survey.

We are interested in understanding the scientific collaborations with investigators at your center as well as with investigators across the ISC^3^ Program. Your participation is voluntary but the information that you provide will help us capture an early benchmark of the scientific linkages that have formed within and across Centers.

Findings from this survey will be shared with NCI, Center PIs/Co-PIs and leadership to help inform the overall program’s efforts to strengthen these ties and support the development of new scientific collaborations to bridge specific areas of research. Further, our hope is that this information will help us identify new approaches to harness these relationships for the advancement of implementation science research.

The survey will take approximately 10-25 minutes to complete. You may choose not to answer any question, and you can exit the survey at any time.

Frequently asked questions about participation and IRB information is located here [web link].

By clicking “Next”, you agree that you have read the above information and are willing to participate.

**[NEXT]**

### Instructions

Thank you for participating in the ISC^3^ Scientific Collaboration Survey!

- If this is your **first time** logging into the survey, click the “A. Frequency of Contact” link in the table below. Links to subsequent sections will become active and clickable as you navigate through the survey.
- To return to this page at any time, click on the “Survey Sections” link at the top of your screen.
- If you are unable to complete the survey in one sitting, you may return to it at another time. Make sure to click **“Save & Exit”** to ensure that the responses you have provided are saved.
- At the end of the survey, you will be able to review your responses and modify them, if needed. Please be sure to click **“Submit”** upon completion.

### Section A. Frequency of Contact

**Please click through all institution headers below to indicate how often you have had direct contact (e.g., meetings, workgroups, phone calls, emails, etc.) with the following individuals *within the last 12 months* (do not count listservs or mass emails). Note that you will not see your name listed below.**

|  | **I do not know this person** | **I know who this person is, but we have had no contact** | **I have had contact with this person at least once in the last 12 months** |
| --- | --- | --- | --- |
| **Oregon Health & Science University** |  |  |  |
| Name 1 |  |  |  |
| Name 2 … |  |  |  |
| **University of Colorado School of Medicine** |  |  |  |
| Name 1 |  |  |  |
| Name 2 … |  |  |  |
| **University of Pennsylvania** |  |  |  |
| Name 1 |  |  |  |
| Name 2 … |  |  |  |
| **University of Washington** |  |  |  |
| Name 1 |  |  |  |
| Name 2 … |  |  |  |
| **Wake Forest School of Medicine** |  |  |  |
| Name 1 |  |  |  |
| Name 2 … |  |  |  |
| **Washington University in St. Louis** |  |  |  |
| Name 1 |  |  |  |
| Name 2 … |  |  |  |
| **Harvard T.H. Chan School of Public Health** |  |  |  |
| Name 1 |  |  |  |
| Name 2 … |  |  |  |
| **National Cancer Institute** |  |  |  |
| Name 1 |  |  |  |
| Name 2 … |  |  |  |

### Section B. Collaborative Activities

**For the individuals you indicated having contact with, please check all of the collaboration activities you have engaged in with them *in the past 12 months*.**

|  | **Planned or conducted research**  (e.g., grant writing, study design or execution) | **Disseminated research to a science audience**  (e.g., scholarly publication, conference presentation) | **Disseminated research to a non-science audience**  (e.g., evaluation report, policy brief) | **Developed products in cross-center work group or committee** (e.g., measures database, survey instrument) | **Engaged in capacity building**  (e.g., trainings, learning communities, mentoring) | **Not Applicable** |
| --- | --- | --- | --- | --- | --- | --- |
| Name1 |  |  |  |  |  |  |
| Name2 |  |  |  |  |  |  |
| Name3 |  |  |  |  |  |  |

**Please list one thing that ISC^3^ (e.g., ISC^3^ centers, cross-center workgroups, NCI) could do that would most enhance scientific collaboration across the 7 funded centers.**

**Are there key research collaborators from the ISC^3^ centers or NCI you feel are missing from the roster in Section A? If so, please provide their names and affiliations:**

### Section C. Background

**In what discipline area does most of your research focus?**

- Public health
- Medicine
- Psychology
- Social work
- Other: _________________________

**How many years of experience do you have in your field of work?**

- Less than 5 years
- 5 to 9 years
- 10-15 years
- More than 15 years

**How would you rate your level of expertise in conducting dissemination and implementation research?**

- Beginner
- Intermediate
- Advanced

**Which of the following best describes your role within the ISC^3^ grant initiative?**

- Doctoral student
- Post-doc
- Staff
- Faculty
- NCI staff
- Other: _________________________

**What best describes your racial/ethnic background?** [check all that apply]

- American Indian or Alaska Native
- Asian
- Black or African American
- Hispanic or Latino
- Native Hawaiian or other Pacific Islander
- White
- Other: _________________________
- Prefer not to answer

**What is your current gender identity?**

- Male
- Female
- Transgender
- Gender non-conforming
- Other: _________________________
- Prefer not to answer

**Thank you for taking the survey**!
