# Supplementary material for: Collaboration networks of the implementation science centers for cancer control: a social network analysis
Source: Implement Sci Commun. 2022 Apr 13;3:41. doi: 10.1186/s43058-022-00290-6 (PMC9009020; doi:10.1186/s43058-022-00290-6)
Supplement: Supplementary file 2 — Additional file 2. ISC3 Year 1 network collaborations. [file 43058_2022_290_MOESM2_ESM.docx]

**Additional File 2.** Implementation Science Centers for Cancer Control (ISC^3^) Year 1 network collaborations (n=192).

|  | **Median (range) degree by collaboration activity**  *# of collaboration ties* | | | | | |
| --- | --- | --- | --- | --- | --- | --- |
| **Characteristic** | **All collaboration activities** | **Planning/ conducting research** | **Capacity building** | **Product development** | **Scientific dissemination** | **Practice/policy dissemination** |
| **Discipline** |  |  |  |  |  |  |
| Public health | 24 (2, 89) | 15 (1, 48) | 12 (1, 58) | 8 (1, 45) | 5 (1, 27) | **3 (1, 21)** |
| Medicine | 21.5 (6, 59) | 17.5 (6, 41) | 9 (2, 53) | 6 (1, 42) | 7.5 (1, 23) | **3 (1, 22)** |
| Other^a^ | 20 (4, 58) | 14 (1, 34) | 9 (1, 44) | 4 (1, 30) | 4 (1, 30) | **2 (1, 10)** |
| **Experience in field** |  |  |  |  |  |  |
| < 5 years | 20 (7, 39) | 14 (4, 26) | 8 (1, 26) | **4 (1, 23)** | **2 (1, 26)** | 1.5 (1, 8) |
| 5-9 years | 24 (2, 54) | 13 (2, 28) | 11 (1, 53) | **4.5 (1, 23)** | **4 (1, 19)** | 2 (1, 14) |
| 10-15 years | 22 (8, 58) | 18 (4, 41) | 9.5 (1, 49) | **5.5 (1, 36)** | **7 (1, 23)** | 2 (1, 22) |
| > 15 years | 24 (4, 89) | 15.5 (1, 48) | 11 (2, 58) | **10 (1, 45)** | **7 (1, 30)** | 3 (1, 21) |
| **Role** |  |  |  |  |  |  |
| Trainee | **19.5 (2, 39)** | **8.5 (2, 21)** | **12.5 (1, 32)** | **2 (1, 5)** | **2.5 (1, 26)** | **2 (1, 5)** |
| Staff | **20 (5, 55)** | **17 (4, 29)** | **7 (1, 48)** | **7 (1, 23)** | **3 (1, 30)** | **4 (1, 22)** |
| Faculty | **24 (4, 89)** | **17 (5, 48)** | **11.5 (1, 58)** | **7.5 (1, 42)** | **7 (1, 27)** | **2 (1, 21)** |
| NCI staff | **28 (6, 65)** | **6 (1, 16)** | **20 (6, 53)** | **7.5 (3, 45)** | **6 (1, 28)** | **1 (1, 2)** |
| Other^b^ | **12.5 (7, 33)** | **11 (4, 14)** | **9.5 (2, 25)** | **4.5 (2, 9)** | **5 (1, 8)** | **1 (1, 6)** |
| **IS expertise level** |  |  |  |  |  |  |
| Beginner | **20 (2, 57)** | **14 (1, 41)** | **7 (1, 49)** | **4 (1, 25)** | **3 (1, 21)** | 2 (1, 18) |
| Intermediate | **20 (4, 55)** | **14 (1, 29)** | **9 (2, 53)** | **5.5 (1, 32)** | **5 (1, 26)** | 2 (1, 22) |
| Advanced | **39.5 (12, 89)** | **20 (5, 48)** | **24 (4, 58)** | **9 (1, 45)** | **12 (1, 30)** | 3 (1, 21) |
| **Gender identity** |  |  |  |  |  |  |
| Female | 24 (5, 65) | 15.5 (1, 41) | 12 (1, 53) | 8 (1, 45) | 6 (1, 30) | 3 (1, 22) |
| Male | 20 (2, 89) | 17 (2, 48) | 8 (1, 58) | 6 (1, 36) | 5 (1, 28) | 2 (1, 21) |
| **Racial/ethnic background** |  |  |  |  |  |  |
| White | **23.5 (4, 89)** | **17 (1, 48)** | 11 (1, 58) | 8 (1, 42) | **7 (1, 30)** | 3 (1, 22) |
| Asian | **18 (2, 65)** | **11.5 (2, 20)** | 6.5 (1, 32) | 5 (1, 45) | **2 (1, 11)** | 2 (1, 5) |
| Black or African American | **13 (5, 50)** | **8 (1, 21)** | 11 (2, 48) | 5 (2, 14) | **2 (1, 9)** | 2.5 (1, 8) |
| Hispanic or Latino | **32 (24, 45)** | **21 (14, 24)** | 19 (10, 27) | 7 (3, 24) | **16 (4, 19)** | 1 (1, 13) |
| Other | **19 (11, 30)** | **6 (4, 18)** | 7.5 (6, 22) | 7 (2, 15) | **6 (4, 9)** | 2 (1, 7) |

IS = implementation science; NCI = National Cancer Institute. Bold values indicate Kruskal-Wallis chi-square test value p<0.05.

^a^ Examples of other disciplines include psychology, social work, economics, health services research, and implementation science.

^b^ Examples of other roles included consultants and advisors.
